# Measurement of flourishing: a scoping review

**DOI:** 10.3389/fpsyg.2024.1293943

**Published:** 2024-02-01

**Authors:** Andrew Rule, Cody Abbey, Huan Wang, Scott Rozelle, Manpreet K. Singh

**Affiliations:** ^1^Stanford Center on China's Economy and Institutions, Stanford University, Stanford, CA, United States; ^2^Stanford School of Medicine, Stanford University, Stanford, CA, United States

**Keywords:** flourishing, measurement, wellbeing, clinical applications, scoping review

## Abstract

**Introduction:**

Flourishing is an evolving wellbeing construct and outcome of interest across the social and biological sciences. Despite some conceptual advancements, there remains limited consensus on how to measure flourishing, as well as how to distinguish it from closely related wellbeing constructs, such as thriving and life satisfaction. This paper aims to provide an overview and comparison of the diverse scales that have been developed to measure flourishing among adolescent and adult populations to provide recommendations for future studies seeking to use flourishing as an outcome in social and biological research.

**Methods:**

In accordance with the Preferred Reporting Items for Systematic Reviews and Meta-Analyses (PRISMA), we conducted a scoping review across PubMed and EMBASE of studies introducing original flourishing scales (defined as a previously unpublished measure of mental health or wellbeing that used “flourishing” in its definition). Studies focusing on adult populations that were published before April 28, 2023 were considered eligible for inclusion.

**Results:**

Out of 781 studies retrieved, we identified seven eligible studies covering seven unique flourishing scales. We find that all seven scales are multidimensional and assess features over monthly or yearly intervals. While most of the scales (six out of seven) include indicators of both hedonic and eudaimonic wellbeing, the operationalization of these dimensions of wellbeing varies considerably between scales. Several of the scales have been translated and validated across multiple geographical contexts, including higher- and lower-income countries.

**Discussion:**

Complementing self-report measures with other social, economic, regional, and biological indicators of flourishing may be useful to provide holistic and widely applicable measures of wellbeing. This review contributes to concept validation efforts that can guide strategies to sustain flourishing societies.

## 1 Introduction

Heightened recent exploration of health and wellbeing has led many to ask what it means to flourish. Indicators of wellbeing are highly complex and often constitute polygenic phenotypes and traits, strongly influenced by the environment, with which an organism interacts in complex ways (Bartels, 2015). Academic disciplines such as preventive medicine, public health, psychology, economics, and other social and biological sciences have shifted in recent years from focusing on economically-focused outcomes of wellbeing, such as per capita income and longevity, toward encompassing a broader range of intrinsic and context-dependent outcomes (Diener and Seligman, 2004; Shrotryia and Singh, 2020; Shiba et al., 2022). Researchers in these fields have increasingly expanded their conception of wellbeing from hedonic aspects (i.e., the presence of positive emotions such as happiness, life satisfaction, quality of life, etc.) to include more eudaimonic aspects of wellbeing (i.e., high levels of functioning across a range of emotions) (Brandel et al., 2017).

The emerging concept of “flourishing” reflects these expanding conceptions of what constitutes holistic wellbeing (VanderWeele et al., 2019; WHO, 2020). The spread of flourishing as a concept used in wellbeing studies can be traced back to Keyes (2002), in which it was defined as having “complete mental health…to be filled with positive emotion and to be functioning well psychologically and socially.” While studies that attempt to measure flourishing have proliferated in the two decades since, no unified definition of flourishing yet exists. Researchers have variously defined flourishing as “a combination of feeling good and functioning effectively” (Huppert and So, 2013), “living the good life” (Seligman, 2012), and “[when] all aspects of a person's life are good…complete human wellbeing” (VanderWeele, 2017). While these definitions share obvious overlap, it is far from clear which components of wellbeing are common to all or nearly all conceptualizations of flourishing, and which components lie closer to the ambiguous conceptual boundaries between flourishing and related wellbeing constructs. For instance, while most conceptualizations of flourishing beginning with Keyes (2002) have included a mix of hedonic and eudaimonic wellbeing indicators, it is not clear whether this is a universal attribute shared by all or nearly all scales designed to measure flourishing. In addition, the overlap between flourishing and related constructs and indicators, a lack of clear instruction on specific applications of flourishing measures, and a relative absence of cultural context all present significant challenges to the formation of a consistently defined and widely understood conceptualization of flourishing.

Addressing these conceptual challenges would be beneficial for both the subfield of flourishing and the broader, often ambiguously defined field of wellbeing as a whole. The poorly or inconsistently defined boundaries between flourishing and other related constructs can make studies that draw on flourishing measures difficult to compare with each other and with the rest of the wellbeing literature. This may hamper the commendable efforts made in recent years to clarify the structure and components of the wellbeing field by meta-analyzing the validity of widely used wellbeing measures. Iasiello et al. (2022), for instance, used data extracted from 26 studies to confirm the validity of the Mental Health Continuum—Short Form, a widely used wellbeing instrument first operationalized in Keyes et al. (2008). Another important attempt to reconcile disparate frameworks within the flourishing literature can be found in Hone et al. (2014), which reviewed the psychometric properties of four existing operationalizations of flourishing and evaluated the agreement between them. However, as these earlier studies do not seek to rigorously identify all existing operationalizations of flourishing, they do not provide insight into the full scope of flourishing as it is currently understood in the literature. To support the concept validation work already initiated by these scholars, it is important to determine which indicators are universally understood to relate to flourishing, and which lie closer to the more ambiguous conceptual boundaries between flourishing and related wellbeing constructs. This type of investigation would lay a foundation for comprehensive future conversations about what outcomes and indicators properly belong within the domain of flourishing, strengthening the cohesion of future flourishing research and helping to clarify the relations between wellbeing concepts within the broader field of wellbeing.

One crucial first step toward resolving these conceptual challenges is identifying, comparing, and reconciling the scales that have been used to measure flourishing in the literature to date. Particularly in the absence of a universal definition of flourishing, a careful accounting of the methodologies and dimensions of existing scales, as well as the extent to which these scales can be compared or supplemented with one another, is crucial to allow flourishing research to be conducted and interpreted across fields. In the past, researchers working in other areas of mental health have similarly turned to reviews of measurement scales to reconcile large literatures covering inconsistently defined concepts. For instance, Dodd et al. (2021) conducted a scoping review of the conceptualization and measurement of mental health among students in the United Kingdom in order to promote more standardized and broadly interpretable mental health measurement in the future. Ong et al. (2021) similarly performed a scoping review to identify and compare 38 existing subjective wellbeing scales, albeit with the goal of informing new scale design in specific contexts rather than facilitating comparison and interpretation across a broader existing field of study. In the field of flourishing, we are only aware of one prior study that has systematically compared the psychometric and conceptual properties of several flourishing scales (Hone et al., 2014). While that study made valuable contributions toward identifying the concordances between widely used operationalizations of flourishing, it considered only four scales and did not use a rigorous scoping review methodology to ensure that all existing flourishing scales were included. To encompass the whole breadth of conceptualizations of flourishing employed in the flourishing literature—including newer scales, less frequently cited scales, and scales designed for specific settings—there is still a need for an updated, rigorous scoping review focused on the literature of flourishing measurement.

We therefore propose that collecting methodological, conceptual, and validation information about existing flourishing scales can aid in the interpretation of past flourishing literature and the production of a more cohesive conceptualization of flourishing in the future. Methodological information allows researchers to compare the sources and formats of various flourishing scales and may include categories such as type of report (e.g., self-report or external evaluation), period in which the described symptoms were experienced, number of items in scale, scoring method (e.g., Likert), measurement invariance (Rosič et al., 2022), or the process used to develop the scale (Weziak-Bialowolska et al., 2021a). Conceptual information reflects the theoretical constructs that the scales are intended to measure and may include the specific definition of flourishing or dimensions of flourishing used in the scale (Agenor et al., 2017; Levin, 2020). Finally, validation information describes the populations (including linguistic contexts, cultural groups, age groups, etc.) for which the scale has been determined valid, and may also include the validation methods and/or variance of the validation study. A snapshot of the populations and contexts in which scales have been validated can help researchers to select appropriate scales for future studies and identify populations that have been under-examined in the literature.

The primary goal of the present study is to identify existing flourishing scales in the literature and examine the aspects in which these scales overlap or diverge, thereby providing more specific indications for use of these scales across the fields of economics, psychology, and medicine. To achieve this, we first conduct a scoping review across various frameworks and operationalizations of flourishing. We then compare scales across properties such as scoring methods, length, development process, and dimensions of flourishing included, highlighting areas of overlap and divergence along the way to guide future applications of and comparisons between the scales. Finally, we provide a snapshot of existing efforts to validate each scale, if any, and make preliminary observations of the populations that have been heavily examined and those that appear to have been overlooked in the literature to date. It should be noted that our review of the literature for flourishing measures is not aimed at judging the quality of scales or prescribing precise applications for extant measures, but rather at contributing to ongoing efforts toward concept/construct validation (Agenor et al., 2017).

## 2 Methods

### 2.1 Search strategy

**Figure 1:**
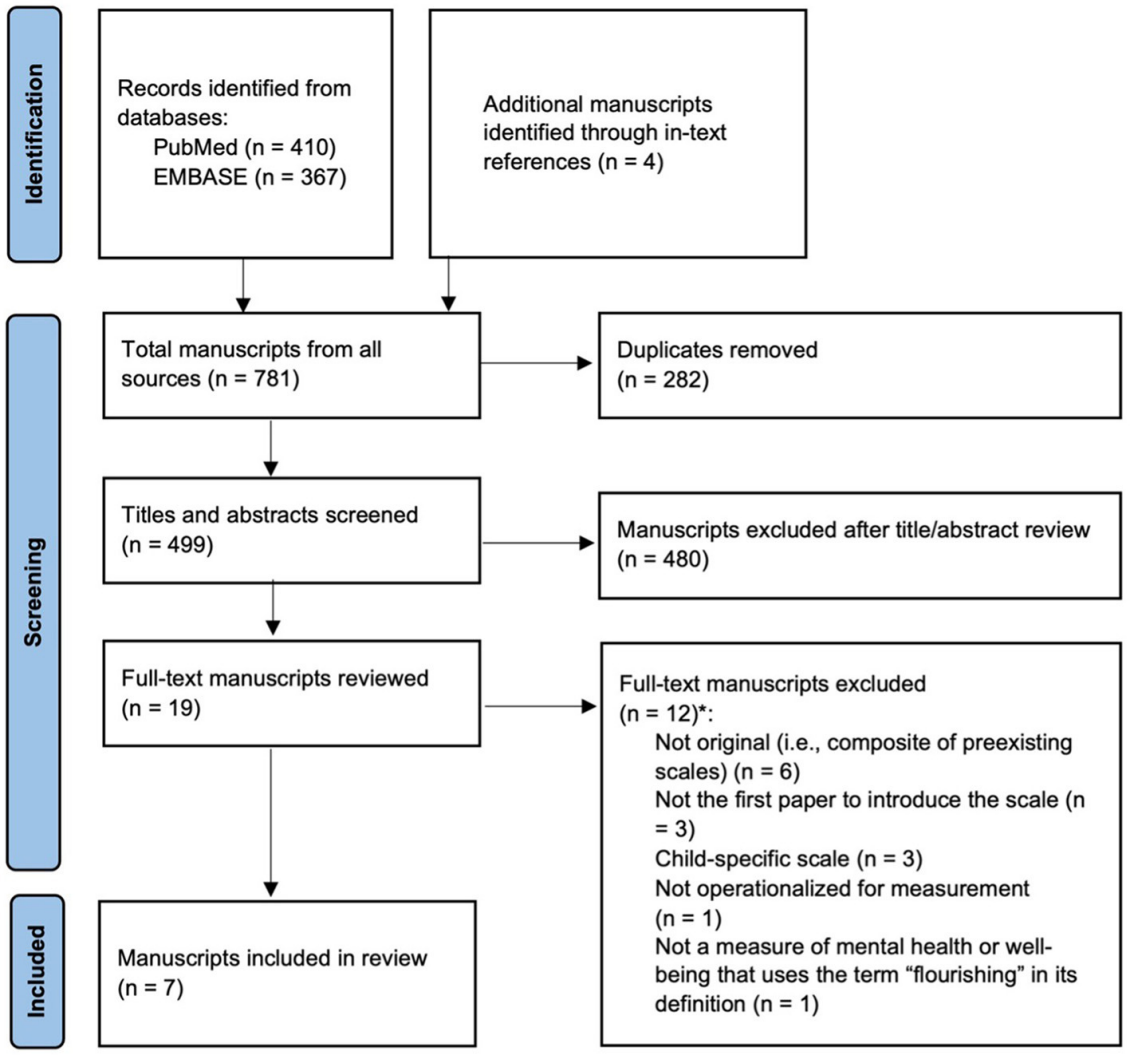
PRISMA flow diagram for scoping review. ^*^Some manuscripts were excluded for multiple reasons.

A scoping review of the literature was conducted in PubMed and EMBASE to identify flourishing scales in eligible studies published on or before April 28, 2023. A research librarian with expertise in conducting scoping reviews assisted in developing a search strategy using terms related to flourishing, which combined concepts related to wellbeing, thriving, and life satisfaction. The following search string was used to search the databases: “flourish^*^[Title/Abstract] AND (well-being[Title/Abstract] OR “mental health”[Title/Abstract]) AND (measure^*^[Title/Abstract] OR scale[Title/Abstract] OR assess^*^[Title/Abstract] OR instrument[Title/Abstract]).” Results were screened independently by two separate reviewers to reduce the risk of bias. When in-text references in screened abstracts referred to potentially eligible flourishing scales whose initial studies were not available on PubMed or EMBASE, these studies were retrieved from Google Scholar and added to the abstract screening pool (see Figure 1).

### 2.2 Eligibility criteria and study selection

Eligibility of papers for inclusion in the review was determined based on the following criteria: (1) The study must present a measure or scale of mental health or wellbeing (including combined physical and mental health) that uses the term “flourishing” in its definition; (2) The study must be the first to introduce the measure or scale; (3) The measure or scale used must be developed for use among adult populations (including scales developed for use among mixed adolescent and adult populations); (4) The measure or scale must be original and not simply a composite of preexisting scales; (5) The measure or scale must be operationalized to measure and compare flourishing (i.e., no theoretical frameworks without instructions for measurement); and (6) The study must have been published in a peer-reviewed academic journal in English before April 28, 2023.

The initial search yielded 777 possible abstracts. Duplicate results were removed, leaving 495 results. Four additional papers were identified through in-text references during the abstract screening stage and were added to the abstract screening pool. A total of 19 papers passed the abstract screening stage and were selected for full-text screening. Finally, after full-text screening, the reviewers identified seven eligible studies containing seven unique flourishing scales (Figure 1). A complete list of studies excluded at the full-text stage and reasons for their exclusion can be found in Supplementary Table 1.

### 2.3 Data extraction

Using a pre-defined template, publication, methodological, and conceptual data about the flourishing scales were extracted from each eligible study. Publication data included the title, authors, date, and publication source of the study. Methodological data included the name of the scale, any alternative scale formats presented in the initial study (e.g., long or short form), type of report (e.g., self, physician, etc.), period over which the scale measures flourishing (past week, past month, etc.), number of scale items, scoring method (e.g., Likert scale), and any details about the process by which the scale was developed. Conceptual data included the definition of flourishing used in the study's introduction and the dimensions of flourishing included in the scale's framework (including subdimensions, if any).

After the final list of eligible studies and flourishing scales was generated, an informal search of validation studies for the scales was conducted on PubMed and Embase to produce a snapshot of the populations and contexts in which the scales have been validated. Validation studies were identified using the search term “{*scale name*}[Title/Abstract] AND valid^*^[Title/Abstract].” Titles and abstracts of the resulting studies were then screened to determine if validation studies of the scale besides its initial study existed. If no other validation studies existed, this was noted. If such validation studies did exist, an anecdotal appraisal of the extent of validation studies was recorded (e.g., fewer than three validation studies, between three and six validation studies, more than six validation studies). Additionally, up to five validation studies per scale were selected for inclusion in the “Global Applications” section of this paper (see **Table 2**), and the region, age, language, and sample size of the population for which the scale was validated were recorded. When more than five validation studies existed, the selection of validation studies for inclusion in the table was made on the basis of geographic and linguistic diversity. While formally reviewing all validation studies conducted for each scale is beyond the scope of this paper, providing an informal snapshot of the breadth of validation for each study in this way allows a rough comparison of the populations in which validated flourishing research across various dimensions has been conducted.

## 3 Results

### 3.1 Dimensions included in flourishing scales: universal and unique variations

Table 1 summarizes the dimensions included in the flourishing scales that we identified in our review. In general, these scales had several commonalities. First, all scales emphasized that flourishing is principally a subjective rather than objective measure of wellbeing, as perceived “from the individual's point of view” (Diener et al., 2010). Second, the scales emphasized that flourishing is a multi-dimensional concept of wellbeing. As pointed out by one study (Huppert and So, 2009), compared to unidimensional outcomes like life satisfaction, which often involve single-item measures, “flourishing is a broader measure with greater texture across different elements.” The studies all mentioned that flourishing involved both psychological and social aspects of functioning (VanderWeele, 2017). These commonalities held across all scales, even those that were only designed to measure flourishing in a specific context, such as the Flourishing-at-Work Scale (Rautenbach and Rothmann, 2017) and the Digital Flourishing Scale (Janicke-Bowles et al., 2023).

**Table 1:** Summary and dimensions of existing flourishing measures.

**Scale name**	**Initial study**	**Type of report**	**Period**	**# of items**	**Scoring method**	**Definition of flourishing**	**Dimensions in framework**
Mental Health Continuum—Short Form (MHC-SF) and Mental Health Continuum—Long Form (MHC-LF)	Keyes et al., 2008^a^	Self	Past month	14 (short) 40 (long)	Likert	“Positive emotions and functioning well psychologically and socially…presence and absence of mental illness and mental health symptoms.”	*Hedonic:* Emotional wellbeing *Eudaimonic:* Self-acceptance, autonomy, personal growth, social integration, social contribution, social actualization
Wellbeing module items on the European Social Survey (“Huppert and So's measure”)	Huppert and So, 2013	Self	In general	10	Likert	“The experience of life going well... a combination of feeling good and functioning effectively. Flourishing is synonymous with a high level of mental well-being, and it epitomizes mental health”	Competence Emotional stability Engagement Meaning Optimism Positive emotion Positive relationships Resilience Self-esteem Vitality
Flourishing scale	Diener et al., 2010^b^	Self	In general	8	Likert	“‘Social-psychological prosperity' from the individual's point of view.”	Social wellbeing Psychological wellbeing (No subscale scores)
PERMA^c^ profiler	Butler and Kern, 2016	Self	In general	23	Likert	“Dynamic optimal state of psychological functioning that arises from functioning well across multiple psychosocial domains.”	Positive emotions Engagement Relationships Meaning Accomplishment (Additional questions on health, loneliness, overall happiness)
Flourishing-at-Work Scale—Short Form (FWS-SF)^d^	Rautenbach and Rothmann, 2017	Self	Past month	17	Likert	“A sense that one's life at work is going well and that one is functioning well”	Emotional wellbeing Psychological wellbeing Social wellbeing
Flourishing Index and Secure Flourishing Index	VanderWeele, 2017	Self	In general	10 (12)	Likert	“All aspects of a person's life are good”; “complete human well-being”	Happiness and life satisfaction Health (mental and physical) Meaning and purpose Character and virtue Close social relationships Financial/material stability (SFI only)
Digital Flourishing Scale	Janicke-Bowles et al., 2023	Self	In general	25	Likert	“Both ‘feeling well' and ‘doing well'... comprises all aspects of well-being, fleeting or lasting, on an experiential as well as a behavioral or functional level”	Connectedness Civil participation Positive social comparison Authentic self-disclosure Self-control

^a^The 40-item MHC-LF was first conceptualized in Keyes (2002), and the 14-item MHC-SF was conceptualized in Keyes (2006) before being first operationalized in Keyes et al. (2008).

^b^The Flourishing Scale was first introduced under the name “Psychological Well-Being” in a book chapter (Diener et al., 2009). The paper cited here is its first use in an academic journal article.

^c^PERMA: positive emotion, engagement, relationships, meaning, and accomplishment.

^d^A 48-item long form of the FWS has been proposed in Rautenbach (2015), but, to the best of our knowledge, it has not yet been used in a peer-reviewed research study.

Notably, all of the studies highlighted that flourishing involves high levels of functioning across a range of emotions (eudaimonia) and is not simply defined by the presence of positive emotions (e.g., hedonia, happiness). This suggests that eudaimonia may be a particularly distinguishing feature of flourishing, possibly serving to differentiate it from other constructs, such as happiness, that instantiate a single positive emotion. Crucially, the majority of the scales (six out of seven) require the presence of both hedonia and eudaimonia for flourishing, echoing the theorization of flourishing in Keyes (2002) as consisting of a mixture of emotional wellbeing (hedonia) and positive functioning (eudaimonia). This suggests that Keyes' theorization is indeed broadly agreed upon among researchers engaged in flourishing scale design and can serve as a foundation for future work to reconcile existing measures. Only one scale, the Flourishing Scale, excluded hedonic wellbeing from its definition (Diener et al., 2010). As such, unlike the other scales we reviewed that measure flourishing holistically, the Flourishing Scale appears to be useful to measure eudaimonic wellbeing only.

While most scales agreed that flourishing consists of both hedonic and eudaimonic wellbeing, the specific ways in which hedonia and eudaimonia are operationalized do differ somewhat between scales. Out of the six scales that measured both types of wellbeing, five specifically selected indicators to measure hedonia or eudaimonia separately, with only the Digital Flourishing Scale (Janicke-Bowles et al., 2023) attempting to account for the presence of both hedonia and eudaimonia using the same set of indicators. Hedonia was generally measured as a single indicator representing happiness/positive affect (Keyes et al., 2008; Huppert and So, 2013; Butler and Kern, 2016; VanderWeele, 2017) or as a pair of indicators representing happiness and unhappiness, respectively (Rautenbach and Rothmann, 2017). The measurement of eudaimonia varied more significantly. The Mental Health Continuum—Short Form (Keyes et al., 2008), Flourishing Scale (Diener et al., 2010), and Flourishing-at-Work Scale—Short Form (Rautenbach and Rothmann, 2017) all divided eudaimonia into psychological wellbeing and social wellbeing. The most common indicators related to psychological wellbeing were meaning or purpose (five scales: Diener et al., 2010; Huppert and So, 2013; Butler and Kern, 2016; Rautenbach and Rothmann, 2017; VanderWeele, 2017); competence or accomplishment (four scales: Diener et al., 2010; Huppert and So, 2013; Butler and Kern, 2016; Rautenbach and Rothmann, 2017); and self-acceptance or self-esteem (three scales: Keyes et al., 2008; Diener et al., 2010; Huppert and So, 2013). As noted by Hone et al. (2014), the Mental Health Continuum—Short Form (Keyes et al., 2008) does not explicitly include competence but does include the closely related construct of environmental mastery. Hone et al. (2014) also recommended that life satisfaction, which was used as an indicator in the Mental Health Continuum—Short Form (Keyes et al., 2008), be adopted for use in other flourishing measures. We find that life satisfaction has indeed been adopted for use in two of the more recent scales we reviewed (Rautenbach and Rothmann, 2017; VanderWeele, 2017), and has been found to correlate strongly with a third recent scale (Janicke-Bowles et al., 2023).

To measure social wellbeing, all scales included at least one item related to positive social relationships (e.g., Huppert and So, 2013; Butler and Kern, 2016; VanderWeele, 2017) or connectedness (Janicke-Bowles et al., 2023). In Keyes et al. (2008), social wellbeing is further decomposed into a list of specific indicators that are more thorough than those used by most other scales but still largely compatible with them, including social integration, contribution, acceptance actualization, and coherence. Finally, a few indicators that appeared in only one or two scales measured qualities that are linked to but not necessarily components of hedonic and eudaimonic wellbeing, including physical health (two scales: Butler and Kern, 2016; VanderWeele, 2017), character and virtue (one scale: VanderWeele, 2017), and financial or material stability (one scale: VanderWeele, 2017). While the relation between these indicators and flourishing is important to clarify in future research, they appear to exist outside of the core concept of flourishing generally held in the scales we reviewed.

Two other characteristics were notably common among the scales. First, few of the scales had “cutoffs” by which they define what is low, average, or high levels of flourishing. This may be due to the highly subjective and individualized nature of such measures and the circumstances that led to their development. As Kern (2021) writes, “different profiles will be best for different people, based on their values, interests, and experiences.” However, the use of cutoffs is still helpful for comparison purposes in the two scales that provide them (Keyes et al., 2008; Huppert and So, 2013), particularly as these scales both also provide continuous measures for flourishing and acknowledge the necessarily arbitrary nature of the cutoffs. Second, the items in the scales almost exclusively asked about the existence of positive outcomes (as opposed to the existence or absence of negative outcomes). To address this, it may be critical to evaluate and balance the valence structure of each item on a flourishing scale, as known attention biases toward more positive or negative valences may moderate individual responses (Thoern et al., 2016).

### 3.2 Scale properties of flourishing measurement

#### 3.2.1 Scale length and scoring

Flourishing scales examined here are psychometrically-based questionnaires designed for use among general adult (or, in one case, working adult) populations. All scales are self-report questionnaires and are scored on a Likert scale (the range of which varies across the scales but generally includes at least five points or options). The scales ask respondents to rate either to what extent they agreed with a statement, how much a statement is relevant to them, or how often they have felt or experienced a certain state or situation. Most asked questions to respondents about their life in general at the time of assessment, with only the Mental Health Continuum—Short Form (Keyes et al., 2008) and the Flourishing-at-Work Scale—Short Form (Rautenbach and Rothmann, 2017) limiting responses to the last month. All scales seem to be designed to be administered no more than once per month. There is some variation in the length of the questionnaires. The shortest is the 8-item Flourishing Scale (Diener et al., 2010), and the longest is the 40-item Mental Health Continuum—Long Form (Keyes, 2002), although the widely-used 14-item short form of this scale has been more commonly used and validated (Keyes et al., 2008).

#### 3.2.2 Scale development

Many scales were developed by applying research theories and traditions to existing surveys that included wellbeing-related measures. For instance, the MHC-SF (Keyes et al., 2008) derives from the 40 items presented in Keyes (2002), which in turn were derived from existing instruments (Ryff, 1989; Keyes, 1998) in the Survey on Midlife Development in the United States (Lamers et al., 2011). Likewise, Huppert and So (2013) selected items from the European Social Survey that they deemed to best correspond to the features of flourishing, from which they created an overall composite score. A factor analysis was recently presented on this flourishing composite (Ruggeri et al., 2020). Butler and Kern (2016) created a pool of 700 items that were either chosen from past surveys or developed *de novo*, and they then systematically rated items based on relevance to the PERMA framework (which stands for the five pillars of wellbeing identified by Seligman—**P**ositive emotion, **E**ngagement, **R**elationships, **M**eaning, and **A**ccomplishment) (Seligman, 2002). Other scales drew items from existing scales measuring related constructs, rather than from existing surveys. Some items in the Flourishing Scale were adapted from Ryff (1989) and Ryan and Deci (2000, 2001), with additional items added to expand the dimensions of flourishing covered by the scale (Diener et al., 2009). VanderWeele's 10-item Flourish Index and 12-item Secure Flourish Index also include items adapted from a variety of previously validated scales and surveys, including the University of Chicago's General Social Survey (University of Chicago, 2017; VanderWeele, 2017). The authors of the Digital Flourishing Scale established the scale's initial 120 items on the basis of self-determination theory after perusing a long list of existing scales, and then shortened the scale to its final 23-item form after a series of confirmatory studies (Janicke-Bowles et al., 2023). Ongoing studies in globally represented populations aim to further enrich the assessment of flourishing possibly as a universally shared human outcome, providing opportunities for broader and unified application of flourishing measurement (Grossmeier, 2017; Weziak-Bialowolska et al., 2021b).

### 3.3 Global applications

Table 2 provides a selection of populations and geographical settings where the scales have been adapted and validated for use. While the table does not exhaustively list all settings and populations for which the scales have been validated, it provides a snapshot of the range of validation for these scales at a moment in time. All scales have been operationalized to measure flourishing in at least one population. However, only five of the seven scales have been validated outside of the study in which they were initially introduced. The scales that have been most frequently and widely validated include the MHC-SF, the Flourishing Scale, and the PERMA Profiler, all of which have been validated in both higher-income countries and lower- and middle-income countries. The full scope of validation efforts for the MHC-SF has been estimated in a recent systematic review and meta-analysis, which identified at least 46 unique studies examining the factor structure of the MHC-SF, covering 103 samples (Iasiello et al., 2022). To our knowledge, the MHC-SF is the only flourishing scale that has been the specific focus of a systematic review in the literature today, reflecting its wide use in international wellbeing research. Moreover, validation studies for the MHC-SF may be published in a wide range of languages, meaning that the scope of validation for this scale may be even greater than indicated in Iasiello et al. (2022). Table 2 presents a fraction of the settings where the MHC-SF has been validated, both higher-income and lower- or middle-income, including the Netherlands (Lamers et al., 2011), South Africa (Keyes et al., 2008), China (Guo et al., 2015), and Argentina (Lupano Perugini et al., 2017). As shown in the table, the Flourishing Scale has also been validated for both higher- and lower-income settings, including but not limited to China (Tong and Wang, 2017), Colombia (Martín-Carbonell et al., 2021), and Iran (Fassih-Ramandi et al., 2020), as well as specific subpopulations including American adults with spinal cord injuries (Perera et al., 2018). The PERMA Profiler was initially more widely used in Western developed countries, but more recently it has been adapted for use in non-Western contexts like Iran (Payoun et al., 2020).

**Table 2:** An international selection of flourishing scale validation studies.

**Scale**	**Validation study**	**Setting**	**Population**	**Sample age**	**Language**	**Sample size**
MHC-SF	Keyes et al., 2008	South Africa	Adults (Black, Setswana-speaking)	30–60	Setswana	1,050
MHC-SF	Lamers et al., 2011	Netherlands	Adults	18–87 (*M* = 48)	Dutch	1,662
MHC-SF	Guo et al., 2015	China	Adolescents (urban areas)	*M* = 15	Chinese	5,399
MHC-SF	Lupano Perugini et al., 2017	Argentina	Adults	*M* = 40	Spanish	1,300
MHC-SF	Żemojtel-Piotrowska et al., 2018	38 nations^a^	University students	16–50	Multiple^a^	8,066
Huppert and So's measure	Huppert and So, 2013	22 European countries^a^	Older adolescents, adults, older adults	15+	Unspecified	43,000
Huppert and So's measure	Ruggeri et al., 2020	21 European countries^a^	Older adolescents, adults, older adults	15–103 (*M* = 48)	Unspecified	41,825
Flourishing scale	Tong and Wang, 2017	Macau (China)	Adults	18–85 (*M* = 40.26)	Chinese	1,008
Flourishing scale	Schotanus-Dijkstra et al., 2016	Netherlands	Adults with suboptimal levels of mental wellbeing	20–67 (*M* = 47.8)	Dutch	275
Flourishing scale	Martín-Carbonell et al., 2021	Colombia	Adults	18–67 (*M* = 26)	Spanish	1,255
Flourishing scale	Perera et al., 2018	U.S.	Adults with spinal cord injury	53.7 (19–93)	English	472
Flourishing scale	Fassih-Ramandi et al., 2020	Iran	Older adults	60-98 (*M* = 66)	Persian	300
PERMA profiler	Watanabe et al., 2018	Japan	Working adults	18+ (*M* = 45.8)	Japanese	310
PERMA profiler	Umucu et al., 2020	U.S.	Student veterans	18-64 (*M* = 30)	English	205
PERMA profiler	Butler and Kern, 2016	16+ countries and regions^a^	ADULTS	18+	Unspecified	31,966
PERMA profiler	Ryan et al., 2019	Australia	Adults	*M* = 41.3	English	439
PERMA profiler	Payoun et al., 2020	Iran	Older adults	*M* = 68	Persian	384
FWS-SF	Rautenbach and Rothmann, 2017	South Africa	Beverage company employees (adults, racially diverse)	“Under 25” to “over 55”	English	779
Flourish index/secure flourish index	Wȩziak-Białowolska et al., 2019	Five nations^a^	Adults	25, 31, 35, 25, 33 (subgroup means)	Unspecified	8,873
Digital Flourishing Scale	Janicke-Bowles et al., 2023	U.S.	Adults	18–88 (*M* = 49)	English	483

^a^The specific settings and languages in which Huppert and So (2013), Butler and Kern (2016), Żemojtel-Piotrowska et al. (2018), Wȩziak-Białowolska et al. (2019), and Ruggeri et al. (2020) were conducted can be found in Supplementary Table 2.

The remaining four scales we identified have been used and validated in many fewer studies than the scales discussed above. While the Flourish Index and Secure Flourish Index (VanderWeele, 2017) have not been as widely validated as the prior three scales, they have nonetheless been used and validated in diverse cultural contexts including China, Sri Lanka, Cambodia, Mexico, and the U.S. (Wȩziak-Białowolska et al., 2019). In contrast, Huppert and So's (2013) scale, although derived from the European Social Survey used in 21 countries, has not been used in contexts outside of Europe (Ruggeri et al., 2020). To our knowledge, the FWS-SF and Digital Flourishing Scale have not been validated outside of their initial studies. Remote and recent validations of flourishing scales across multiple settings and countries illustrate the broad adaptability of the measurement of flourishing (Fowers et al., 2016; Tong and Wang, 2017; Romano et al., 2020; Martín-Carbonell et al., 2021; Rosič et al., 2022).

## 4 Discussion

In this scoping review, we have identified and compared the existing scales that have been developed to measure flourishing across a variety of contexts and academic disciplines. Numerous conceptual problems with the construct of flourishing remain, including the lack of a unified definition of flourishing; the need for clarity in the links between flourishing, mental health, and mental illness; the proliferation of overlapping wellbeing constructs in the literature; and the difficulty of comparing flourishing research results across scales. While the present review cannot resolve these issues, it is hoped that the analysis presented here can guide researchers through the landscape of the flourishing literature as it currently exists and offer a first step toward a more cohesive body of flourishing research. Below, we consider the insight that our findings can provide into these conceptual challenges, as well as the next steps that will be needed to resolve them.

As our review has shown, flourishing measures have several characteristics that, together, distinguish them from measurements of other wellbeing constructs. Flourishing scales are generally subjective measurements of wellbeing, differentiating them from other quality-of-life scales that ask individuals to objectively assess the availability of resources in their environment (Weziak-Bialowolska et al., 2021a). Flourishing scales are also multi-dimensional in nature, with several items and individual subscale scores to measure such dimensions. This makes them different from most life satisfaction measures and some happiness measures, which are often limited to a single item or a series of single-item dimensions, that are ultimately also multidimensionally determined (Jovanović and Lazić, 2020; Singh et al., 2023). Relatedly, flourishing scales all emphasize functioning in multiple aspects of everyday life (eudaimonia), including psychological, social, and—for certain scales—physical functioning. Flourishing items are also designed to be administered at relatively long intervals (no more frequently than once per month), suggesting they are designed to indicate a summative or sustained period of wellbeing rather than a measure of wellbeing that is only valid at a single time point or following an adverse event.

There are several other commonalities across flourishing scales that have implications for their application in research and policy. More than half of the flourishing measures have been translated into multiple languages and validated for use in a range of geographical contexts (including nations of both higher and lower income levels for some scales). Several have also been applied with psychometric properties examined across the lifespan, ranging from adolescent to older adult populations. In addition, the dimensions of wellbeing measured by flourishing scales are relatively consistent compared with other wellbeing constructs, as most include dimensions or several items related to social relationships, meaning (or purpose), life satisfaction/happiness, mastery (or accomplishment), and engagement. Further, flourishing scales tend to be relatively short (usually not exceeding 25 items in length) and scored on a Likert scale. These characteristics provide some support for a universal conceptualization of flourishing that can be practically implemented in a variety of contexts with good uptake.

We also observe some ways in which flourishing scales differ from one another as they have evolved. Despite the presence of common dimensions across scales, others, such as optimism, health (physical/mental), social coherence/acceptance, financial/material stability, morality/virtue, positive/negative emotions, resilience, and loneliness, were measured by only a single scale or by a minority of scales. Newer scales have incorporated dimensions explicitly considered distinct from flourishing by past authors, like health. Incorporating health outcomes appears to be a likely trend in flourishing measurement, as two of the more recent flourishing scales both encompassed items related to mental and physical health (Butler and Kern, 2016; VanderWeele, 2017). This is exemplified by the recent proposal in Kern (2022) that the PERMA framework, the basis for one of the flourishing scales analyzed in this paper, be adjusted to PERMA**H** so as to include a **h**ealth dimension. Morality and virtue are other dimensions covered in one of the more recent scales (VanderWeele, 2017) that were not considered previously. These differences across scales and variations in how scales are evolving pose potential challenges for validation and universal conceptualization.

Incidentally, our review also reveals the salience of debates about the precise relation between mental wellbeing, mental health, and mental illness for the measurement of flourishing. These debates have given rise to two opposing models of mental health in the literature in recent decades. Some scholars have proposed that mental health is structured as a single continuum from symptoms of mental illness to happiness and wellbeing (Wood and Tarrier, 2010). Other scholars propose instead a two-continua model, in which a patient exists simultaneously on a mental health continuum running from severe symptoms of mental illness to no symptoms of mental illness, and on a wellbeing continuum running from low to high wellbeing (Tudor, 1996; Keyes, 2002). The lack of agreement between proponents of the one-continuum and two-continua models is reflected in the scales we reviewed. Some of the scales adhere generally to a two-continua model of mental health, epitomized by the Mental Health Continuum (MHC) (Keyes, 2002; Keyes et al., 2008), which places flourishing on a spectrum representing the absence/presence of complete mental health (as distinct from diagnosable mental illness). However, even among the scales that use a two-continua model of mental health, there is variation in how these continua are conceptualized, with Butler and Kern (2016) and Janicke-Bowles et al. (2023) placing flourishing on a separate continuum from mental health and mental illness, and VanderWeele (2017) positing that mental health is only a small component of a larger construct known as flourishing. Other studies, namely the Flourishing-at-Work Scale (Rautenbach and Rothmann, 2017) and Huppert and So's (2009) scale, mark mental illness and flourishing as the lowest and highest points on a single spectrum, indicating adherence to the one-continuum model. The Flourishing Scale (Diener et al., 2010) is unique in that it draws no explicit link between flourishing and mental health or mental illness. It is clear that efforts to form a cohesive conceptualization of flourishing should be informed by these ongoing debates in the broader mental health literature.

Even beyond concept validation efforts, how mental health and flourishing are related has implications for nosology and for developing novel treatment strategies. For example, treatment endpoints in psychiatric clinical trials focus on symptom outcomes that may be unrelated to overall wellbeing in individuals who suffer from serious mental illness (Braslow and Marder, 2019). Wellbeing may be a more acceptable and durable outcome than immediate symptom relief. Moreover, a focus on symptoms and pathology may be stigmatizing (Oexle et al., 2017), whereas wellbeing may be less stigmatizing. Acceptance and impact of this kind of shift merits further study.

Recently, flourishing scales (particularly the Secure Flourishing Index in VanderWeele, 2017) have experimented with the idea of including measures of individuals' environments, such as financial security and material wellbeing. It is difficult to form conclusions about trends in flourishing scale revision and development based on limited implementation. However, these adaptations suggest a move toward considering one's access to resources that allow for the realization of wellbeing in other aspects of life. VanderWeele (2017) argues that such resources are “not…ends in themselves but may be important in the preservation of those goods that are their own ends.” In other words, to sustain flourishing across other dimensions, a certain amount of access to financial and material assets may be essential. In sum, flourishing measures have evolved to become increasingly comprehensive by incorporating more aspects of an individual's wellbeing in the context of a broader social framework.

Our review also underscores the importance of evaluating eudaimonic and social dimensions of wellbeing (Gallagher et al., 2009) alongside hedonic dimensions of wellbeing that are self-reported, to assess flourishing comprehensively. There is a temptation to collate several positively-valenced outcomes together, especially when positive outcomes correlate with each other (Sirgy, 2019). However, when a eudaimonic-focused flourishing scale was tested for validity in individuals with low to moderate levels of wellbeing, positive skewness of the scale resulted in lower precision at higher bounds of the social-psychological continuum, limiting its external validity and use in intervention studies or in clinical practice (Schotanus-Dijkstra et al., 2016). A complementary assessment of hedonic and eudaimonic dimensions, with varying positive and negative valence items, and which is assessed over time to examine the temporal stability and dynamics of a flourishing scale, may provide a more comprehensive and reliable treatment of this outcome than has been previously presented.

Indeed, indicators of flourishing cohere along biological, psychological, and social dimensions that may well vary widely, evolve, or even have limited utility for repeated measurement or used in the context of an intervention. Network-based approaches to provide a data-driven heuristic on how latent classes of wellbeing might relate to one another have been recently reported (Giuntoli and Vidotto, 2021), though the general heterogeneity of different indicators of wellbeing preclude a global or summative assessment of flourishing outcome or assignment of use case. Acknowledging these limitations, a variety of composite flourishing scales and indices result in total scores that may have practical applications in health (Faul et al., 2018) and policy (Trudel-Fitzgerald et al., 2019) if applied thoughtfully and with appreciation of the type of information they convey.

Besides issues related to measurement and scale design, there are other general directions for future flourishing research. First, measures of child and adolescent flourishing, like the non-child/adolescent measures examined in this review, cover a broad variety of dimensions with limited consistency across scales and would benefit from a dedicated scoping review and evaluation. Relatedly, few if any studies have examined how flourishing varies across different stages of life. Evaluating wellbeing dimensions early in life and repeatedly across the lifespan may enable prediction of flourishing outcomes for subjects living in a range of geographic contexts. This may even include those who migrate from one geographic context to another—an important population to take into account, as residential mobility is an important risk factor for emotional and behavioral difficulties beginning in childhood (Jelleyman and Spencer, 2008; Nikolof et al., 2023). Four pathways may be central to determining one's flourishing across different dimensions of life—family, work, school, and community (VanderWeele, 2017)—and these pathways likely vary in their relevance at different life stages and may not be exhaustive. Factors most strongly associated with subjective wellbeing can even vary across zip codes in the same county (Chrisinger et al., 2019). Second, future studies should continue exploring if and how flourishing can be used as an intervention outcome (Feicht et al., 2013; Gimpel et al., 2014; Ahmad et al., 2020; Chilver and Gatt, 2022). Indeed, many psychological interventions have been systematically evaluated for improving mental wellbeing in general (van Agteren et al., 2021) and in the workplace (Sakuraya et al., 2020). However, approaches to testing the efficacy and long-term durability of interventions aimed not only at improving symptoms in the short term but also wellbeing in the longer term remain undeveloped. As patient-centered outcomes become more central to mental health interventions, and as we learn to better define clinically *meaningful* outcomes, understanding and refining flourishing measurement is timely in mental health care. Third, the scales we reviewed typically conceptualize flourishing as a relatively long-term construct (i.e., needing to be measured no more than once per month), indicating that scales are intended not to be susceptible to short-term or state-dependent fluctuations in happiness or life satisfaction. Examining the stability of flourishing as a construct in different contexts and stages of life is an important direction for future flourishing assessments. More broadly, comprehensive assessments of the long-term impact of positively valenced emotional states that includes tracking of individual biological or social contributors of wellbeing across the lifespan would also be beneficial. Finally, although flourishing scales will always be imperfect measures of complexity in the real world and there may be no one right model (Box, 1976; Kern, 2021), we can still strive to identify the frameworks and tools that are most helpful for specific contexts, which we appreciate may vary significantly based on individual identities, backgrounds, and migration patterns.

We acknowledge a few limitations of our evaluation of the extant literature on flourishing measurement. First, our initial search took place in only two databases (PubMed and EMBASE) that focus on biomedical and psychological research. While we made efforts to conduct the most comprehensive search possible, including conducting additional screenings of in-text references in the included abstracts to improve the reach of our search, it remains possible that some relevant studies may not have been included, particularly in fields outside of psychology or medicine. Second, comprehensive validation information might properly be considered outside the scope of a rigorous review of flourishing scales, as it is drawn from validation studies rather than from the original studies introducing the scales. Third, it is increasingly clear that cross-cultural frameworks are needed to consider the context in which flourishing is measured. We are encouraged to see several scales that have recently been validated in cross-cultural settings to support a hypothesis that flourishing may be a universal construct that can be cross-culturally contextualized (Żemojtel-Piotrowska et al., 2018; Santini et al., 2020). Any adaptations, however, may make it challenging to harmonize the wellbeing construct across societies.

## 5 Conclusion

This study has reviewed the literature on flourishing as a wellbeing outcome, including general patterns and variations in how flourishing has been measured and applied. Existing flourishing measures appear to share several commonalities (including multi-dimensionality, self-reporting of subjective wellbeing, an emphasis on everyday functioning, and relative stability across time) that together make them unique from other wellbeing constructs, though flourishing scales are also evolving their own dimensions. We also outlined directions for future research, including broadening the methods used to design and validate these scales, exploring their use in clinical settings, reviewing and evaluating flourishing scales for use among pre-adolescents and adolescents, and assessing how the latent constructs of flourishing relate to and predict one another over the course of the lifespan. As flourishing becomes increasingly salient for individuals, communities, and policymakers, its measurement may become increasingly context-dependent, and its application may be increasingly relevant as an intervention outcome.

## Data availability statement

The original contributions presented in the study are included in the article/Supplementary material. Further inquiries can be directed to the corresponding author.

## Author contributions

AR: Conceptualization, Investigation, Methodology, Visualization, Writing – original draft, Writing – review & editing. CA: Conceptualization, Methodology, Writing – original draft, Writing – review & editing. HW: Conceptualization, Investigation, Validation, Writing – review & editing. SR: Conceptualization, Supervision, Writing – review & editing. MS: Conceptualization, Investigation, Methodology, Project administration, Writing – original draft, Writing – review & editing.
