# Supplementary material for: Measurement of flourishing: a scoping review
Source: Front Psychol. 2024 Feb 1;15:1293943. doi: 10.3389/fpsyg.2024.1293943 (PMC10867253; doi:10.3389/fpsyg.2024.1293943)
Supplement: Supplementary file 1 [file Table_1.DOCX]

**Supplementary Table 1: Flourishing Scale Studies Excluded at Full-Text Screening Stage**

| **Scale name** | **Study** | **Reasons for exclusion** |
| --- | --- | --- |
| n/a | Venning et al. (2013) | - Must be a measure of mental health or well-being (including combined physical and mental health) that uses the term “flourishing” in its definition - Must be the first paper to introduce the scale |
| COMPAS-W | Gatt et al. (2014) | - Must be original (i.e., no composites of preexisting scales) |
| Flourishing Scale (shortened) | Conner et al. (2017) | - Must be the first paper to introduce the scale |
| Flourishing items from the National Survey of Children’s Health | Ruest et al. (2018) | - Must be developed for use among adolescents and/or adult populations (no child-specific scales) |
| Flourishing items from European Social Survey | Warr (2018) | - Must be operationalized for measurement (i.e., no theoretical frameworks without instructions for measurement) - Must be the first paper to introduce the scale |
| n/a | Chen et al. (2019) | - Must be original (i.e., no composites of preexisting scales) |
| Flourishing Index | A. C. Faul et al. (2019) | - Must be original (i.e., no composites of preexisting scales) |
| Child Flourishing Index | Clark et al. (2020) | - Must be developed for use among adolescents and/or adult populations (no child-specific scales) |
| Well-Being Assessment | Weziak-Bialowolska, Bialowolski, Lee, et al. (2021) | - Must be original (i.e., no composites of preexisting scales) |
| n/a | Burns et al. (2022) | - Must be original (i.e., no composites of preexisting scales) |
| n/a | Whitaker et al. (2022) | - Must be developed for use among adolescents and/or adult populations (no child-specific scales) |
| n/a | Wydick et al. (2022) | - Must be original (i.e., no composites of preexisting scales) |
