# Supplementary material for: Measurement of flourishing: a scoping review
Source: Front Psychol. 2024 Feb 1;15:1293943. doi: 10.3389/fpsyg.2024.1293943 (PMC10867253; doi:10.3389/fpsyg.2024.1293943)
Supplement: Supplementary file 2 [file Table_2.DOCX]

**Supplementary Table 2: Settings and Languages of Validation Studies Conducted in Multiple Countries**

| **Scale** | **Validation study** | **Setting** | **Sample size** | **Language** |
| --- | --- | --- | --- | --- |
| MHC-SF | Zemojtel-Piotrowska et al. (2018) | ﻿Algeria | 240 | Arabic |
|  |  | Armenia | 223 | Armenian |
|  |  | Azerbaijan | 120 | Russian |
|  |  | Belgium | 232 | Flemish |
|  |  | Brazil | 223 | Portuguese |
|  |  | Bulgaria | 200 | Bulgarian |
|  |  | Chile | 241 | Spanish |
|  |  | Colombia | 138 | Spanish |
|  |  | Czech Republic | 223 | Czech |
|  |  | Estonia | 301 | Esti |
|  |  | Germany | 233 | German |
|  |  | Hong Kong | 172 | English |
|  |  | Hungary | 206 | Hungarian |
|  |  | India | 200 | English |
|  |  | Indonesia | 200 | Bahasa |
|  |  | Iran | 201 | English |
|  |  | Japan | 195 | Japanese |
|  |  | Kazakhstan | 285 | Russian |
|  |  | Kenya | 162 | English |
|  |  | Korea (S) | 212 | Korean |
|  |  | Latvia | 221 | Russian |
|  |  | Malaysia | 199 | Malay |
|  |  | Nepal | 203 | English |
|  |  | Panama | 170 | Spanish |
|  |  | Pakistan | 200 | English |
|  |  | Poland | 227 | Polish |
|  |  | Portugal | 193 | Portuguese |
|  |  | Puerto | 300 | Spanish |
|  |  | Romania | 206 | Romanian |
|  |  | Russia | 229 | Russian |
|  |  | Serbia | 205 | Serbian |
|  |  | Slovakia | 202 | Slovak |
|  |  | Spain | 196 | Spanish |
|  |  | South Africa | 186 | English |
|  |  | Ukraine | 171 | Russian |
|  |  | United Kingdom | 303 | English |
|  |  | Uruguay | 197 | Spanish |
|  |  | Vietnam | 251 | Vietnamese |
| Huppert & So’s measure | F. A. Huppert & So (2013) | Denmark | Total 43,000 | Unspecified (all languages in each country for that are the primary language of at least 5% of the population) |
|  |  | Finland |  |  |
|  |  | Norway |  |  |
|  |  | Sweden |  |  |
|  |  | Austria |  |  |
|  |  | Belgium |  |  |
|  |  | Cyprus |  |  |
|  |  | France |  |  |
|  |  | Germany |  |  |
|  |  | Ireland |  |  |
|  |  | Netherlands |  |  |
|  |  | Portugal |  |  |
|  |  | Spain |  |  |
|  |  | Switzerland |  |  |
|  |  | United Kingdom |  |  |
|  |  | Bulgaria |  |  |
|  |  | Estonia |  |  |
|  |  | Russian Federation |  |  |
|  |  | Poland |  |  |
|  |  | Slovakia |  |  |
|  |  | Slovenia |  |  |
|  |  | Ukraine |  |  |
| Huppert & So’s measure | Ruggeri et al. (2020) | Denmark | Total 41,825 | Unspecified (all languages in each country for that are the primary language of at least 5% of the population) |
|  |  | Finland |  |  |
|  |  | Norway |  |  |
|  |  | Sweden |  |  |
|  |  | Belgium |  |  |
|  |  | Cyprus |  |  |
|  |  | France |  |  |
|  |  | Germany |  |  |
|  |  | Ireland |  |  |
|  |  | Netherlands |  |  |
|  |  | Portugal |  |  |
|  |  | Spain |  |  |
|  |  | Switzerland |  |  |
|  |  | United Kingdom |  |  |
|  |  | Bulgaria |  |  |
|  |  | Estonia |  |  |
|  |  | Russian Federation |  |  |
|  |  | Poland |  |  |
|  |  | Slovakia |  |  |
|  |  | Slovenia |  |  |
|  |  | Ukraine |  |  |
| PERMA Profiler | Butler & Kern (2016) | United States | 15,345 | Unspecified |
|  |  | United Kingdom | 1,793 |  |
|  |  | Canada | 1,785 |  |
|  |  | Central/South America | 805 |  |
|  |  | Western Europe | 1,162 |  |
|  |  | Northern Europe | 476 |  |
|  |  | Southern/Eastern Europe | 500 |  |
|  |  | Middle East | 205 |  |
|  |  | Africa | 329 |  |
|  |  | India/Southeast Asia | 1,009 |  |
|  |  | Asia | 508 |  |
|  |  | Australia/New Zealand | 3,270 |  |
|  |  | Other | 2,406 |  |
| Flourish Index / Secure Flourish Index | Wȩziak-Białowolska et al. (2019) | United States | 4,083 | Unspecified |
|  |  | Sri Lanka | 1,284 |  |
|  |  | Cambodia | 587 |  |
|  |  | China | 419 |  |
|  |  | Mexico | 2,500 |  |
